# Supplementary material for: Association between oral microbiome and five types of respiratory infections: a two-sample Mendelian randomization study in east Asian population
Source: Front Microbiol. 2024 Apr 10;15:1392473. doi: 10.3389/fmicb.2024.1392473 (PMC11039966; doi:10.3389/fmicb.2024.1392473)
Supplement: Supplementary file 1 [file Data_Sheet_1.zip › Supplementary Materials/MR plots of saliva/Tonsillitis/s__Rothia_sp001808955_mgs_25/forest.pdf]

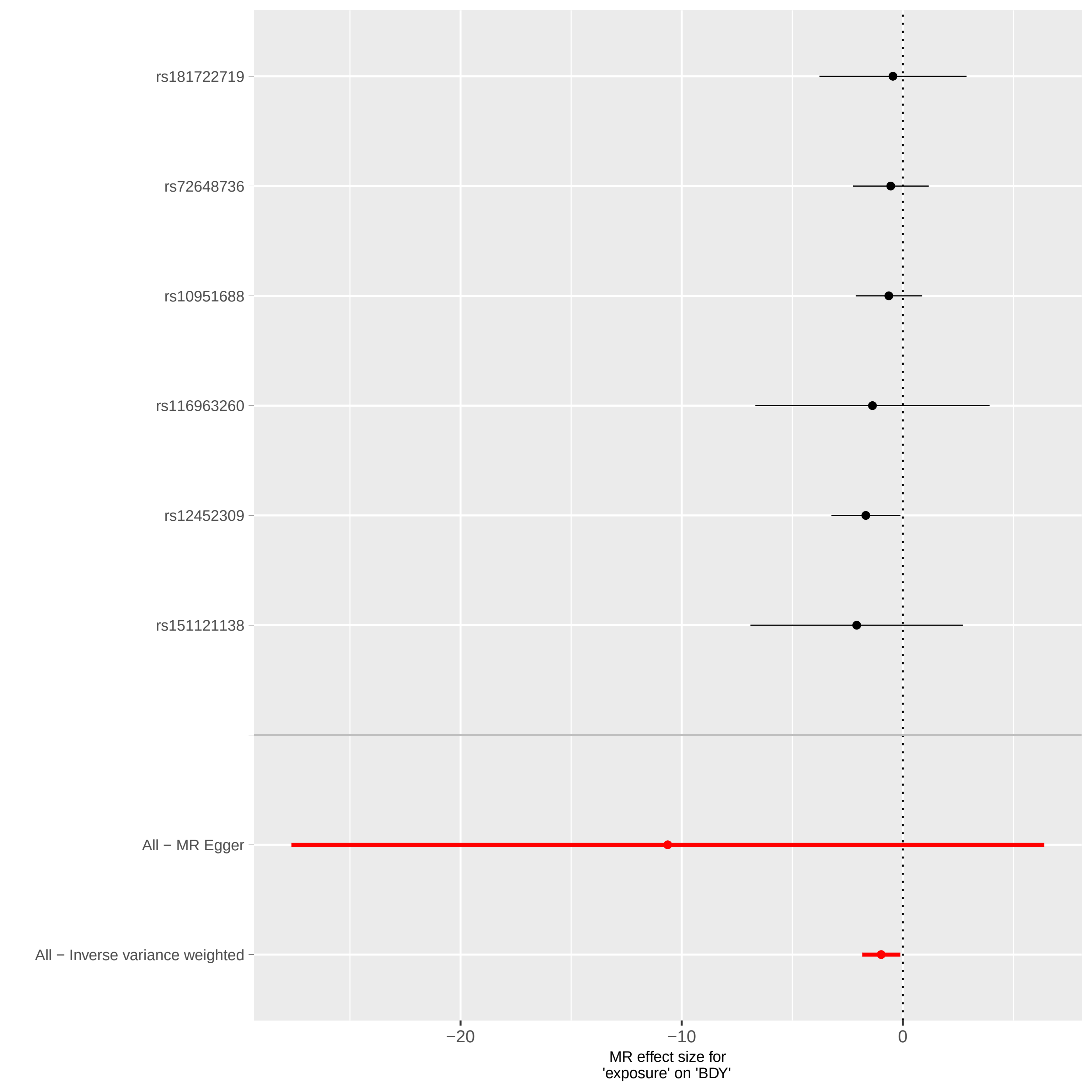

rs181722719
rs72648736
rs10951688
rs116963260
rs12452309
rs151121138
All − MR Egger
All − Inverse variance weighted
−20
−10
0
MR effect size for
'exposure' on 'BDY'
